# Current Pharmacological Management of Gastroesophageal Reflux Disease

**DOI:** 10.1155/2013/983653

**Published:** 2013-06-26

**Authors:** Yao-Kuang Wang, Wen-Hung Hsu, Sophie S. W. Wang, Chien-Yu Lu, Fu-Chen Kuo, Yu-Chung Su, Sheau-Fang Yang, Chiao-Yun Chen, Deng-Chyang Wu, Chao-Hung Kuo

**Affiliations:** ^1^Division of Gastroenterology, Department of Internal Medicine, Kaohsiung Medical University Hospital, Kaohsiung, Taiwan; ^2^Division of Internal Medicine, Kaohsiung Municipal Hsiao-Kang Hospital, Kaohsiung Medical University, Kaohsiung, Taiwan; ^3^Cancer Center, Kaohsiung Medical University Hospital, Kaohsiung, Taiwan; ^4^Department of Medicine, Faculty of Medicine, College of Medicine, Kaohsiung Medical University, Kaohsiung, Taiwan; ^5^Department of Health Management, I-Shou University, E-Da Hospital, Kaohsiung, Taiwan; ^6^Department of Internal Medicine, Kaohsiung Municipal Ta-Tung Hospital, Kaohsiung, Taiwan; ^7^Department of Pathology, Kaohsiung Medical University Hospital, Kaohsiung, Taiwan; ^8^Department of Medical Imaging, Kaohsiung Medical University Hospital, Kaohsiung, Taiwan

## Abstract

Gastroesophageal reflux disease (GERD), a common disorder with troublesome symptoms caused by reflux of gastric contents into the esophagus, has adverse impact on quality of life. A variety of medications have been used in GERD treatment, and acid suppression therapy is the mainstay of treatment for GERD. Although proton pump inhibitor is the most potent acid suppressant and provides good efficacy in esophagitis healing and symptom relief, about one-third of patients with GERD still have persistent symptoms with poor response to standard dose PPI. Antacids, alginate, histamine type-2 receptor antagonists, and prokinetic agents are usually used as add-on therapy to PPI in clinical practice. Development of novel therapeutic agents has focused on the underlying mechanisms of GERD, such as transient lower esophageal sphincter relaxation, motility disorder, mucosal protection, and esophageal hypersensitivity. Newer formulations of PPI with faster and longer duration of action and potassium-competitive acid blocker, a newer acid suppressant, have also been investigated in clinical trials. In this review, we summarize the current and developing therapeutic agents for GERD treatment.

## 1. Introduction

Gastroesophageal reflux disease (GERD) is a common gastrointestinal disorder in the general population, and its prevalence is increasing worldwide [1]. According to the Montreal definition, GERD is diagnosed when the reflux of stomach contents causes troublesome symptoms and/or complications [2], and it is the most common outpatient gastrointestinal disease diagnosed in USA [3]. Reflux from stomach causes symptoms like heartburn and regurgitation, which are the cardinal symptoms of GERD, and other symptoms, such as chest pain, asthma, hoarseness, and sleep disturbance, are also considered as atypical or extraesophageal symptoms of GERD [4]. Troublesome symptoms of GERD have adverse impact on health-related quality of life (HRQL) [5], and patients with more frequent or more severe symptoms have lower HRQL, work productivity, and sleep quality [5, 6]. Chronic reflux is also an important risk factor of esophageal adenocarcinoma [7].

There are many factors contributing to GERD, including transient lower esophageal sphincter relaxation (TLESR), reduced LES pressure, impaired esophageal mucosal defense, poor esophageal clearance, visceral hypersensitivity, hiatal hernia, and delayed gastric emptying, and TLESRs is the predominant mechanism of reflux formation [8]. Obesity is an independent risk factor for development of GERD and is also associated with its complications, including erosive esophagitis, Barrett's esophagus, and esophageal adenocarcinoma [9, 10]. Acid pocket is a short zone of unbuffered highly acidic gastric juice after meal. Discovery of acid pocket has been helpful in understanding postprandial acid reflux and has an influence on management strategies [11, 12]. Both erosive esophagitis and nonerosive reflux disease (NERD) are included in GERD, and the difference between them is whether mucosal damage is detected by endoscopy or not. Patients with NERD have increased sensitivity to weakly acidic or nonacid reflux and abnormal peripheral and central sensitizations resulting in symptoms in these patients [13].

Acid suppression is the mainstay of therapy for GERD and proton pump inhibitors (PPIs) are the most potent drug in this regard. Although the use of PPIs is the treatment of choice for GERD, still approximately one-third of patients with GERD fail to response symptomatically to a standard dose PPI, either partially or completely [14, 15]. Refractory GERD, defined as reflux symptoms either completely or incompletely responsive to PPI therapy, has become an important issue in clinical practice. Treatment options, such as histamine type-2 receptor antagonist (H2RA), TLESR reducers, prokinetic agents, and alginates, could be considered as an add-on to PPI therapy for symptomatic patients after taking PPI. Newer drug and other therapeutic strategies targeting mechanism of GERD, other than acid suppression, are also being developed for patients with incomplete response to PPI. In this review, we summarize the current and developing therapeutic options for GERD treatment:

 Antacids
 Alginate
 Sucralfate
 Acid suppressants
   Histamine type-2 receptor antagonist
   Proton pump inhibitor
   Potassium-competitive acid blocker
 TLESR reducers
   GABA_B_ receptor agonist
   mGluR5 antagonist
 Prokinetic agents
   Metoclopramide
   Domperidone
   Tegaserod
   Mosapride
   Itopride
   Rikkunshito
 Pain modulators
   Tricyclic antidepressants
   Trazodone
   Selective serotonin reuptake inhibitors
   Serotonin-norepinephrine reuptake inhibitor
   Theophylline.

## 2. Therapy Focused on Antacids and Alginate

### 2.1. Antacids

Before H2RA development, antacids were widely used as initial treatment for patient with reflux symptoms. Antacids are compounds containing different combinations, such as calcium carbonate, sodium bicarbonate, aluminum, and magnesium hydroxide. They provide rapid but short-term symptom relief by buffering gastric acid. Antacids are a convenient over-the-counter treatment for GERD, but only one-quarter of patients have symptom relief after antacid use. Nevertheless, these drugs have no efficacy in healing erosive esophagitis [16].

### 2.2. Alginate

Alginate is anionic polysaccharide occurring naturally in brown algae and has a unique property different from traditional antacids. Alginate and bicarbonate, usually contained in alginate-based formulations, interact with gastric acid to form a foamy gel, and this foamy gel, like a raft floating on the surface of gastric contents, creates a relative pH-neutral barrier [17]. Alginate-antacid formulations can reduce postprandial symptoms by neutralizing the acidity of gastric contents and, more importantly, by forming a gel-like barrier to displace the “acid pocket” from the esophagogastric junction and protect the esophageal mucosa [18]. Like antacids, alginate-based formulations demonstrate an immediate onset of effect within 1 hour of administration, faster than PPI and H2RA [19]. Furthermore, alginate-based formulations have longer duration [17] and higher efficacy than traditional antacids in relieving reflux symptoms, even in NERD patients [20]. The mechanism of symptom relief in NERD patients treated with alginate is possibly related to protection of esophageal mucosal integrity [21]. The other potential role of alginate in GERD patients is reducing the damaging of nonacid reflux, like pepsin and bile acids [22]. A randomized double-blind double-dummy trial in moderate GERD patients showed that an alginate-based formulation, Gaviscon (4 × 10 mL/day), was noninferior to omeprazole (20 mg/day) in achieving a 24 h heartburn-free period [23]. Although alginate has less benefit in healing erosive esophagitis [24], it could be considered as an alternative or add-on therapy for symptom relief in GERD patients refractory to PPI [25].

## 3. Therapy Focused on Mucosal Protection

### 3.1. Sucralfate

Sucralfate, a complex salt of sucrose sulfate and aluminum hydroxide, contributes to mucosal protection by several different actions. It provides a physical barrier to block diffusion of acid, pepsin, and bile acids across esophageal mucosa and attenuate the erosive injury of acid and alkali. The potential benefits of sucralfate include mucosa repair and ulcer healing [26]. Sucralfate shows its efficacy in improving reflux symptoms in patients with reflux esophagitis and NERD patients [27, 28]. Like antacids and alginate, sucralfate has a limited role in healing of erosive esophagitis and is usually considered as add-on therapy for GERD treatment. For its low maternal adverse events and no teratogenicity, sucralfate is a safe drug for pregnant woman with reflux symptoms [29].

## 4. Therapy Focused on Acid Suppression

### 4.1. Histamine Type-2 Receptor Antagonist (H2RA)

Before development of PPIs, H2RAs were the first acid-suppressive agents and have better efficacy than antacids in healing of erosive esophagitis and alleviating reflux symptoms. H2RA reduces gastric acid output as well as gastric acid volume by competitive inhibition of histamine at H2 receptors and reducing pepsin secretion. However, patients with severe erosive esophagitis have poorer therapeutic response to H2RA, and most patients with GERD have only improved, but not eliminated, reflux symptoms after H2RA use. H2RAs also have their limitations in treating erosive esophagitis, such as their relatively short duration of action (compared with PPIs), development of tolerance, and incomplete inhibition of acid secretion in response to a meal [30]. In meta-analysis, H2RAs are less effective than PPIs in healing of erosive esophagitis and reliving heartburn [31, 32]. 

Although H2RAs are not as effective as PPI in acid suppression, the potential effect of H2RAs on the nighttime histamine-driven surge in gastric acid secretion makes H2RAs an add-on therapy for patients with nighttime symptoms on PPI treatment such as nocturnal acid breakthrough (NAB). NAB is defined as a gastric pH < 4 for a period greater than 1 hour overnight in patients on twice-daily PPI therapy and occurs in more than 70% of patients on PPI therapy [33]. Addition of a nighttime H2RA to twice-daily PPI can reduce the percentage of NAB and lead to an improvement of nighttime reflux symptoms and sustained efficacy in short-term and long-term use [34, 35]. There are no significant differences between different H2RA agents in suppressing gastric acid, and different H2RAs are considered to have equivalent efficacy. At present, H2RAs are still popular over-the-counter medicines and widely used for controlling GERD symptoms because of their rapid onset of action [36].

### 4.2. Proton Pump Inhibitor (PPI)

PPI blocks the gastric H^+^/K^+^-adenosine triphosphatase (ATPase) via covalent binding to cysteine residues of the proton pump to inhibit gastric acid secretion and is the most potent type of acid suppressants nowadays. Inhibition of H^+^/K^+^-ATPase is more effective than antagonism of H2R in suppressing gastric acid secretion because H^+^/K^+^-ATPase is the final step of acid secretion. Several trials and reviews have shown that PPIs are more effective in healing of erosive esophagitis and symptomatic relief than H2RAs [31, 37–39]. Eighty-three percent of patients with GERD symptoms and 78% of patients with erosive esophagitis have response to PPI treatment [40]. Many studies have evaluated the efficacy or superiority between different PPIs (esomeprazole, lansoprazole, pantoprazole, and rabeprazole) and, the results were inconsistent [41, 42].

Although PPI is the most successful acid suppressant in the treatment of GERD, unsatisfactory results still exist during PPI therapy. Fifty-nine percent of GERD patients with long-term PPI therapy still have persistent reflux symptoms [43]. About one-third of patients fail to adequately response to PPI therapy, and different groups of GERD, like erosive esophagitis, NERD, and Barrett's esophagus, have different response rates to PPI. NERD patients demonstrate the lowest response rate to PPI, and PPI symptomatic response rate in NERD patients is only about 50–60% [43]. The definition of PPI failure is controversial, and refractory GERD is a term used to describe incomplete esophageal healing and/or unsatisfactory symptomatic response after a full course of PPI treatment. The mechanisms of failure of PPI therapy are complicated and multifactorial [44, 45]:

 Non-reflux-related causes
   Esophageal motility disorder, like achalasia,
   scleroderma
   Other esophagitis, like eosinophilic, pill, infection
   Functional heartburn or functional chest pain
 Reflux-related causes
   Compliance
   Rapid PPI metabolism (CYP2C19 polymorphisms)
   Nocturnal acid breakthrough
   Gastric acid hypersecretory states, like Zollinger-Ellison syndrome
   Anatomic abnormality, like large hiatal hernia
   Delayed gastric emptying
   Weakly acidic reflux
   Duodenogastroesophageal (bile) reflux
   Impairment of esophageal mucosal integrity
   Esophageal hypersensitivity
   Psychological comorbidity, like depression, anxiety, life stress
   Concomitant functional bowel disorder.

Traditional PPIs (omeprazole, lansoprazole, pantoprazole, rabeprazole, and esomeprazole) have relatively slow onset of action and provide insufficient 24-hour suppression of gastric acid under a once-daily dosage regime. Novel PPIs have been designed to improve the PPI efficacy with the advantage of rapid onset of action, extended-released profile, and longer half-life.

Tenatoprazole is a novel PPI characterized by an imidazopyridine ring in place of the benzimidazole moiety found in other proton pump inhibitors. Tenatoprazole has longer plasma half-life in comparison with other PPIs, providing a prolonged duration of acid inhibition and a shorter nocturnal acid breakthrough [46, 47]. Even though the plasma half-life of tenatoprazole is about seven times longer than that of other PPIs, tenatoprazole is considered a good alternative PPI for patients with ineffective once-daily PPI therapy [48]. However, the real efficacy of tenatoprazole on patients with GERD needs further investigation because most clinical trials have been performed in healthy volunteers. On the other hand, dexlansoprazole MR is a modified release formulation of dexlansoprazole and has a unique dual delayed-release formulation, which results in a dual-peak time-concentration profile as opposed to the single peak seen with conventional PPIs. The dual delayed-release technology, made by two types of granules containing Dexlansoprazole MR capsule, provides two distinct drug-release periods in the small intestine, which extends plasma drug concentrations and prolongs the therapeutic time [49]. In previous reviews, dexlansoprazole MR has shown its greater effect in healing of erosive esophagitis, maintenance of esophagitis healing, and relief of symptoms in NERD patients as compared with traditional delay-released (DR) PPI [50, 51]. However, the therapeutic potential of dexlansoprazole MR in refractory GERD patients needs further evaluation. The other potential benefits of dexlansoprazole MR used in GERD patients include greater dosing flexibility without regard to meals, effective control of nocturnal heartburn and GERD-related sleep disturbances, and less drug-drug interaction with clopidogrel as compared with omeprazole or esomeprazole [52–54]. A single-blind, multicenter study which enrolled patients taking twice-daily PPI for heartburn control evaluated the efficacy of once-daily dexlansoprazole MR 30 mg as a step-down therapy for twice-daily PPI. This trial demonstrated that heartburn remained well controlled in 88% of patients after step-down to once-daily dexlansoprazole MR 30 mg. However, this study did not compare the efficacy between once-daily dexlansoprazole MR and once-daily traditional PPI as step-down therapy in this patient group [55].

Traditional PPIs are DR PPI because they are acid-labile and need enteric coating to prevent degradation in the stomach, resulting in relatively slow onset of pharmacological action. Traditional PPIs require several doses to achieve adequate acid suppression but fail to achieve adequate 24-hour acid suppression, allowing nocturnal acid breakthrough. Unlike DR PPI, immediate-release (IR) omeprazole is a formulation of nonenteric-coated omeprazole combined with sodium bicarbonate, which protects omeprazole from degradation by gastric acid, and is characterized by more rapid onset of antisecretory action compared with DR PPIs. Based on administration time, IR omeprazole provides profound control of postprandial and nocturnal intragastric acidity. The faster action of IR omeprazole is not influenced by concomitant antacid or food, which attenuates the efficacy of traditional DR PPI on acid suppression [56]. A randomized study conducted in GERD patients with nocturnal symptoms showed that bedtime dosing of IR omeprazole provided significant faster control of nighttime gastric pH and decreased nocturnal acid breakthrough compared with esomeprazole and lansoprazole. IR omeprazole also provided better nocturnal gastric acid control than lansoprazole and comparable efficacy with esomeprazole, suggesting that immediate-release omeprazole may be useful in treating nighttime heartburn [57]. IR omeprazole also provides adequate control of daytime gastric acidity compared with traditional PPIs. Howden et al. evaluated 24-hour intragastric acidity in GERD patients treated with once-daily IR omeprazole and found that morning dosing of IR omeprazole achieved better control of 24-hour intragastric acidity than lansoprazole and pantoprazole [58]. Buffered esomeprazole is another IR formulation and is an oral preparation consisting of an inner core of nonenteric-coated esomeprazole. Buffered esomeprazole achieved significantly faster control of intragastric acidity and higher 24-hour median intragastric pH compared with pantoprazole in healthy volunteers [59]. The advantages of buffered esomeprazole use in GERD patients need further evaluation.

Extended-release (ER) rabeprazole is designed to provide initial acid suppression similar to DR PPI and maintain the plasma exposure of PPI over a longer period, achieving sufficient duration of acid suppression over a 24-hour period. Each ER rabeprazole formulation contains a single rabeprazole enteric-coated DR tablet and multiple rabeprazole pulsatile-release tablets, with prolonged pharmacodynamics effect performed by releasing rabeprazole in the intestine and colon separately. A study conducted in healthy volunteers showed that once-daily ER rabeprazole demonstrated a significantly longer gastric acid suppression (mean percentage of time with gastric pH > 4) over a 24-hour period compared with esomeprazole 40 mg and standard DR rabeprazole 20 mg, and formulations containing 50 mg ER rabeprazole showed the best pharmacodynamics profile compared with other dosages [60]. ER rabeprazole 50 mg once daily is as effective as esomeprazole 40 mg once daily in healing moderate-to-severe erosive esophagitis and heartburn resolution in a combined analysis of two studies, and the subgroup analysis suggests a better healing rate of severe esophagitis in an ER rabeprazole group [61].

VECAM is a combination of a PPI and succinic acid (an acid pump activator that has the same acid-stimulating activity as pentagastrin) and has a meal-independent antisecretory effect. Coadministration of succinic acid with PPI resulted in augmented PPI effects in animal models. A recent study that evaluated efficacy of once-daily VECAM and omeprazole in healthy volunteers showed that VECAM was significantly better in maintaining intragastric pH > 4 during the nighttime than omeprazole 20 mg, which may provide a therapeutic gain in nocturnal symptom control [62].

Long-term use of PPI as maintenance treatment raises the concern of long-term safety of PPI use. Several studies suggest that PPI use may be associated with osteoporotic fractures, enteric infections, community-acquired pneumonia, benign fundic gland polyps, malabsorption of calcium, magnesium, vitamin B12, and iron and decreasing efficacy of clopidogrel. However, most of these results came from observation in epidemiologic case-control studies, and many confounders may contribute to these associations. To date, the evidence of serious side effects from long-term PPI use is poor, and absolute risk of complications attributed to PPIs is low [63, 64].

### 4.3. Potassium-Competitive Acid Blocker (P-CAB)

Potassium-competitive acid blockers (P-CABs) are another class of acid suppressants developed in the last few years and inhibit proton pumps via a different mechanism than PPIs. By competing with binding of the potassium-binding site of proton pump, P-CABs reversibly inhibit gastric H^+^/K^+^-ATPase and do not require acidactivation, which means that they are mealtimeindependent in contrast to PPIs. P-CAB is absorbed very quickly and provides rapid and profound acid suppression by achieving peak plasma concentration rapidly. Several P-CABs such as revaprazan (YH1885), soraprazan, and AZD0865 have been evaluated in animal model and healthy volunteers, and these results have suggested that this group of acid suppressive drugs has a much faster onset of action and may provide greater acid suppression than conventional PPIs [65–67]. However, initial clinical trials with AZD0865 did not show better results than conventional PPI in GERD treatment. In treatment of erosive esophagitis, AZD0865 once daily only provided similar efficacy to esomeprazole 40 mg once daily in healing and controlling symptoms of erosive esophagitis [68]. In another clinical trial of AZD0865 and esomeprazole for the treatment of patients with NERD, AZD0865 also failed to demonstrate better heartburn control than esomeprazole in patients with NERD [69]. Liver toxicity was also observed in several P-CABs during early stages of drug development.

TAK-438 is a new type of P-CAB developed recently and has a slower dissociation rate from proton pumps than other P-CABs by higher pKa. In animal studies, TAK-438 showed a more potent and longer-lasting antisecretory effect than lansoprazole and other P-CABs [70–72].

## 5. Therapy Focused on TLESR

TLESRs are defined as periods of spontaneous, simultaneous relaxation of the lower esophageal sphincter and crural diaphragm. Reflux of gastric content during TLESRs causes reflux symptoms, and TLESRs are the main mechanism of all types of gastroesophageal reflux, including acid and nonacid reflux episodes [73]. TLESRs are primarily triggered by gastric distension through a vagovagal reflex initiated by activation of mechanoreceptors in the cardiac of stomach [74]. Several pharmacologic agents, including nitric oxide synthase inhibitors, cannabinoid agonists (CB1 receptor agonists), cholecystokinin receptor 1 (CCK1) antagonists, *γ*-aminobutyric acid type B (GABA_B_) receptor agonists, and metabotropic glutamate receptor 5 (mGluR5) antagonists, have been developed as TLESR reducers. However, some of these compounds did not provide clinically relevant effect and demonstrated undesirable pharmacologic side effects in clinical trials. At present, only GABA_B_ receptor agonists and mGluR5 antagonists have reached the stage of clinical use and are the most promising agents of TLESR reduction [75].

### 5.1. GABA_**B**_ Receptor Agonists

GABA_B_ receptors are located at many sites within the central and peripheral nervous systems. GABA, as a major inhibitory neurotransmitter within the central nervous system, controls TLESRS by GABA_B_ receptors expressed in LES-projecting neurons of the vagal nerve and the subnucleus centralis of the nucleus tractus solitarius. Other than effect from central nuclei, peripheral GABA_B_ receptors also have inhibitory effect on gastric vagal mechanoreceptors and gastric distention-related TLESRs [76].

Baclofen, usually used in the management of spasticity, is a prototypical GABA_B_ agonist and has effects in the control of TLESRs, initially noted in animal and healthy human studies [77, 78]. In patients with GERD, baclofen significantly decreases the number of reflux events and reflux symptoms by reducing the incidence of TLESRs [79–81]. The effect of baclofen is also seen in patients with hiatal hernia [79]. In addition to control of acid reflux, baclofen also has inhibitory effect on nonacid and duodenal reflux as well as associated symptoms, suggesting a potential role of baclofen as add-on treatment in the management of refractory GERD [82, 83]. In recent studies, baclofen is also effective in attenuating extraesophageal symptoms of GERD. A study of patients with nighttime heartburn showed that baclofen reduced the number of reflux events during sleep and significantly improved sleep quality [84]. In a case series study enrolling three patients with refractory chronic cough due to GERD and being nonrsponsive to PPI, baclofen 20 mg three times a day was given to substitute for domperidone and the cough was resolved after a 2–4-week course of baclofen in all patients [85]. Although baclofen is a promising agent of GABA_B_ agonists in the management of GERD, the routine usage of baclofen in clinical practice is limited because of poor tolerability due to central nervous system-related side effects, such as weakness, drowsiness, confusion, dizziness, headache, and trembling. In an attempt to overcome these limitations, other GABA_B_ agonists, such as arbaclofen placarbil or lesogaberan have been developed to improve tolerability.

Arbaclofen placarbil is an actively transported prodrug of the active R-isomer of baclofen and is efficiently absorbed throughout the intestine and colon, which allows it to be developed in a sustained release formulation. Arbaclofen placarbil has lower dosing frequency and more stable plasma concentration compared with baclofen to improve the safety profile [86]. A study to evaluate arbaclofen placarbil as monotherapy in 44 patients with GERD demonstrated that arbaclofen placarbil 60 mg once daily significantly decreased the number of reflux episodes and number of reflux-associated heartburn events over a period of 12 hours compared with placebo. Arbaclofen placarbil also provides a favorable tolerability and safety profile in this study [87]. However, arbaclofen placarbil was not superior to placebo in relieving heartburn in a subsequent randomized, double-blind, placebocontrolled trial of 156 patients with GERD [88]. Recently, no further studies with arbaclofen placarbil in GERD have been reported, and further development of this agent seems to be stopped.

Lesogaberan, a GABA_B_ agonist that does not cross the blood-brain barrier and mainly acts on peripheral GABA_B_ receptors, is designed to overcome the side effects of baclofen. In healthy volunteers, lesogaberan significantly reduces the number of TLESRs by 36% and acid reflux episodes by approximately 44% and increases LES pressure by 39% compared with placebo [89]. These effects are also found in patients with reflux symptoms despite PPI treatment and lesogaberan being well tolerated [90]. Based on successful results mentioned above, lesogaberan was evaluated as an add-on to PPI therapy in patients with persistent GERD symptoms despite receiving PPI therapy in the following two double-blinded, placebo-controlled, randomized studies. In a phase IIa study with a total of 244 randomised patients, 232 adult patients (114 lesogaberan- and 118 placebo-treated) received either lesogaberan (65 mg twice daily) or placebo in addition to PPI therapy for a period of 4 weeks and were analyzed for efficacy. Treatment with lesogaberan, compared with placebo, resulted in increasing proportion of responders from 8% to 16% and increasing proportion of symptom-free days from 23% to 37% in heartburn and from 25% to 38% in regurgitation [91]. A recent dose-finding phase IIb study was conducted in 661 patients with partial response to PPI therapy, and persistent GERD symptoms demonstrated that lesogaberan at a dose 240 mg twice daily in addition to PPI was found to achieve a statistically significant response compared with placebo (26.2% versus 17.9%, *P* < 0.1). The major side effect noted in this study was reversible elevated alanine transaminase levels (1.1%) [92]. The aforementioned studies demonstrate a relatively modest therapeutic effect of lesogaberan, yet this is insufficient for lesogaberan to be considered as a treatment option for refractory GERD. Further development of this compound was terminated.

### 5.2. mGluR5 Antagonists

Glutamate is the primary neurotransmitter involved in signalling from visceral and somatic primary afferents to the central nervous system. Peripherally located mGluR5 receptors have been associated with control of TLESRs, noted by animal studies initially, and mGluR5 antagonists are considered as potential therapy for patient with GERD [93].

ADX10059 is a potent selective negative allosteric modulator of the mGluR5 and is the most extensively studied agent of mGluR5 antagonists. In the first proof-of-concept study, two groups of 12 patients with GERD demonstrated ADX10059 250 mg three times daily significantly reduced esophageal acid exposure and symptomatic reflux episodes and were welltolerated [94]. A modified release (MR) formulation of ADX10059 had been tested in healthy volunteers, and ADX10059 MR 125 mg twice daily significantly decreased postprandial weakly acidic reflux episodes and esophageal acid exposure [95]. In a larger randomized clinical trial involving 103 patients with GERD, ADX10059 120 mg twice daily as monotherapy for 2 weeks significantly increased GORD symptom-free days and heartburn-free days, reduced antacid use, and improved total symptom score compared with placebo. ADX10059 was well tolerated and common adverse events in this study were mild-to-moderate dizziness and vertigo [96]. Despite good safety and tolerability in these short-term trials, further development of ADX10059 has been halted because of high incidence of adverse hepatic effects in a large multicenter trial of ADX10059 in migraine patients.

AZD2066 is a novel elective, noncompetitive antagonist of mGluR5 and has been studied in healthy volunteers. In a randomized crossover study, AZD2066 significantly reduced TLESRs and reflux episodes in healthy volunteers and had acceptable safety and tolerability profile [97]. The efficacy of AZD2066 in the management of GERD needs further investigation.

## 6. Therapy Focused on Gastroesophageal Motility

Function of gastroesophageal motility is an important factor influencing the pathophysiology of GERD, and disordered gastroesophageal motility includes reduced LES pressure, ineffective esophageal motility, and delayed gastric emptying [98]. Prokinetic agents are a heterogenous class of compounds acting on different receptors, including 5-hydroxytryptamine_4_ (5-HT_4_) receptor agonists, dopamine_2_ (D_2_) receptor antagonists, and motilin and ghrelin receptor agonists, and these compounds are proposed to improve GERD symptoms by enhancing esophageal motility and gastric emptying. However, prokinetic agents are usually not highly selective and provide off-target effects, which lead to controversial therapeutic benefits and undesirable side effects. Metoclopramide (D_2_ antagonist), domperidone (dopamine antagonist), cisapride (5-HT_4_ agonist) and tegaserod (5-HT_4_ agonist) were usually used in patients with GERD in the past, but routine use of these agents was not suggested by guidelines because of limited benefits and high side-effect profile [40]. Erythromycin and ABT-229 are motilin receptor agonists, which are proposed to accelerate gastric emptying and increase LES pressure, and are still not routinely used as prokinetics in GERD because of several limitations [99]. Prokinetic agents are usually used in combination with acid suppression agents as an adjunctive, rather than as sole treatment of GERD.

### 6.1. Mosapride and Itopride

Mosapride, a prokinetic with selective 5-HT_4_ receptor agonist and weak 5-HT_3_ receptor antagonist actions, is effective in reducing acid reflux in the esophagus by improving esophageal motility and gastric emptying. Furthermore, mosapride is well tolerated and no serious adverse events are reported [100]. Mosapride is less effective than PPI as monotherapy in the management of GERD and is usually used as an adjunct to PPI therapy. Coadministration of mosapride has favorable influence on pharmacokinetics of PPI by accelerating the absorption of PPI and increasing maximum plasma concentration and the area under the time-plasma concentration curve and combination therapy with mosapride and PPI increases intragastric pH more rapidly than using PPI alone [101, 102]. However, mosapride as add-on therapy to PPI in patients with erosive esophagitis fails to provide better symptom relief than placebo, and additional benefits of mosapride are only possibly seen in patients with severe symptoms [103]. A double-blind, placebocontrolled study with mosapride in NERD patients demonstrated that addition of mosapride to PPI was not more effective than placebo in improving reflux symptoms [104]. In another study investigating efficacy of mosapride as add-on therapy to omeprazole in PPI-resistant NERD patients, improving reflux symptoms and gastric emptying was found in patients with delayed gastric emptying [105]. A recent small study showed that the addition of mosapride to esomeprazole improved esophageal peristaltic function in patients with GERD, but treatment response was not different between mosapride and placebo groups. Moreover, in the same study, better response seemed to be found in patients with dyspepsia than in those without dyspepsia [106]. Mosapride may provide additional benefit as add-on therapy in some special groups like those with motility disorder, rather than the general population.

Itopride, a D_2_ antagonist with anticholinesterase activity, accelerates gastric emptying through both antidopaminergic and antiacetylcholinesterase actions. It is usually used in the treatment of patients with functional dyspepsia and has good efficacy in postprandial fullness and early satiety. A pilot study conducted in 26 patients with GERD symptoms showed that itopride 100 mg three times a day improved GERD symptoms and decreased esophageal acid exposure, and no serious adverse events were noted [107]. However, recent mechanistic studies demonstrated that itopride had no significant influence on gastric emptying, esophageal peristaltic function, and LES pressure. Therapeutic benefit of itopride may come from influence on brain-gut correlation, visceral hypersensitivity, gastric accommodation, distension-induced adaptation, and TLESRs [108, 109]. Itopride has also been used in patients with laryngopharyngeal reflux as an add-on therapy to PPI for extraesophageal symptoms, but itopride did not provide better efficacy than placebo, only accelerated improvement rate [110, 111].

### 6.2. Rikkunshito (TJ-43)

Rikkunshito, a traditional Japanese medicine, is composed of eight crude herbs and is widely used in Japan for patients with various gastrointestinal symptoms such as anorexia, nausea, and vomiting. Rikkunshito ameliorates the effects of nitric oxide-mediated gastric function to improve gastric emptying; besides, it also increases ghrelin levels, a potent stimulant for gastric emptying and gastrointestinal motility [112]. Rikkunshito reduced distal esophageal acid exposure by improving esophageal acid clearance in a small study conducted in children with GERD [113]. In healthy volunteers, standard dose Rikkunshito has no significant influence on postprandial acid or nonacid reflux events and does not accelerate esophageal clearance time [114]. In a study with Rikkunshito as combination therapy with rabeprazole (10 mg/day) in patients with refractory GERD showing resistant symptoms after a 4-week course of rabeprazole, combination therapy had similar efficacy of symptom relief compared with double-dose rabeprazole. In this study, subgroup analysis demonstrated that combination therapy was more effective than double-dose PPI in male patients with NERD [115]. Furthermore, Rikkunshito has strong binding capacity of bile salts and adsorption of bile salt, giving it a potential role in the management of refractory GERD related to duodenogastroesophageal reflux, which deserves further evaluation [116].

## 7. Therapy Focused on Visceral Hypersensitivity

Visceral hypersensitivity has been suggested to be an important mechanism of refractory GERD in patients with NERD and functional heartburn. The pathophysiology of esophageal hypersensitivity is complex, and visceral hypersensitivity resulting from upregulation of nociceptive pathways by peripheral and central sensitization and psycho neuroimmune interactions is proposed. Heightened perception threshold and response function for stimulus within physiology range, like weakly acidic, nonacidic, or bile reflux, cause chest pain, heartburn, or reflux symptoms in these patients [117, 118]. Furthermore, psychological comorbidity also influences GERD symptom burden and treatment response to PPI [119]. Tricyclic antidepressants, trazodone, and selective serotonin reuptake inhibitors have been used as pain modulators to improve esophageal pain in patients with noncardiac chest pain [120]. Serotonin-norepinephrine reuptake inhibitor and theophylline also improve esophageal hypersensitivity in patients with functional chest pain [121, 122]. Although these pain modulators are used in low non-mood-altering doses, side effects are relatively common. At present, these visceral analgesics provide a therapeutic alternative for PPI failure patients as add-on therapy or monotherapy [120].

Transient receptor potential vanilloid 1 (TRPV1) is a polymodal receptor, sensitive to noxious heat, change in pH (acidosis and alkalosis), endovanilloids, and numerous pungent plant products such as capsaicin, piperine, and eugenol, and it can be both upregulated and sensitized during inflammation and injury via peripheral and central nervous pathways. Studies have demonstrated that TRPV1 is a critical channel for mediating thermal hyperalgesia from noxious heat stimulation in mice, and these results have generated great interest in developing TRPV1 antagonists as pain modulators [123]. AZD1386 is a new TRPV1 antagonist and currently under investigation for esophageal pain in humans. In healthy men, AZD1386 reduces the threshold of esophageal pain perception in response to heat, but not to acid, mechanical, or electrical stimulation, as compared with placebo. A rise in body temperature and feeling cold reported by volunteers were observed in an AZD1386 group in this study [124]. Another study with AZD1386 in NERD patients with insufficient response to PPI demonstrated that AZD1386 did not significantly change pain threshold for heat, mechanical or electrical stimulation [125].

## 8. Pharmacological Options for Refractory GERD

The mechanisms of refractory GERD are complicated, and clarification of the possible causes of PPI failure is important to deal with these patients. Compliance to therapy should be checked first by physician, and the presence of functional gastrointestinal disorders, psychological distress, functional heartburn, or other esophagitis not related to reflux should also be carefully evaluated in these patients.

With some proven benefits, switching to another PPI or doubling the PPI dose has become the most common therapeutic strategy for patients who failed PPI once-daily treatment in clinical practice. When prescribing high-dose PPI, the dose is given twice daily before breakfast and dinner to have better control of intragastric pH [45, 126]. Although new formulations of PPIs can provide more immediate, potent, or consistent acid suppression, the real efficacy of newer PPIs for refractory GERD is still limited. Alginate and H2RA provide additional benefit on symptom relief in patients with persistent symptoms despite PPI therapy and can be considered as add-on therapy for refractory GERD [25, 35]. Under the concern of tolerance, H2RA is suggested to be taken on demand or intermittently. Baclofen is the most promising agent of TLESR reducer, but routine use in patients with refractory GERD is not favored because of neurological side effects. Mosapride may provide additional benefit as add-on therapy in patients with severe symptoms or gastroesophageal motility disorder [103, 105]. Rikkunshito is a potent prokinetic and can be used as add-on therapy to PPI [115]. The value of pain modulators in the management of refractory GERD needs further evaluation.

## 9. Conclusion

To date, PPIs are still the most effective therapeutic tool and should be suggested as mainstay of treatment in patients with GERD. If symptoms continue despite adequate PPI use, the poor compliance or inadequate dosing time should be excluded before diagnosing refractory GERD in patients with poor response to PPI. The causes of refractory GERD are complex, and symptoms from weakly acidic or nonacid reflux suggest that acid suppression cannot be the only solution for all patients with GERD. New PPI formulations and new acid suppressants, P-CABs, have not shown clinical superiority to current PPIs. Nevertheless, newer PPI formulations with longer duration of action provide additional benefit in patients with poor compliance or nocturnal symptoms. In addition to PPI, TLESR reducers have been considered as the most promising strategies in the management of GERD. However, the therapeutic gain of TLESR reducers observed in patients with GERD was relatively small. Prokinetics have potential role as add-on therapy to PPIs and may provide additional benefit in special groups. Pain modulators that attenuate esophageal hypersensitivity are in the early phase of development, and the efficacy as well as tolerability needs further investigation. Overall, the target population for these new therapeutic agents remains to be defined by future studies. Despite the well-established benefits of current PPIs in the management of GERD, unmet needs are still present and require further pharmacologic development to provide viable options for better GERD treatment.
